# The role of p19 and p21 H-Ras proteins and mutants in miRNA expression in cancer and a Costello syndrome cell model

**DOI:** 10.1186/s12881-015-0184-z

**Published:** 2015-07-03

**Authors:** Roseli García-Cruz, Maria Camats, George A. Calin, Chang-Gong Liu, Stefano Volinia, Cristian Taccioli, Carlo M. Croce, Montse Bach-Elias

**Affiliations:** Instituto de Investigaciones Biomédicas de Barcelona- CSIC, C/ Egipcíacas15, 08001 Barcelona, Spain; Ohio State University, Department of Molecular Immunology, Virology and Molecular Genetics, Columbus, Ohio 43210 USA; Present address: Departments of Experimental Therapeutics & Cancer Genetics, University of Texas, MD Anderson Cancer Center, Houston, TX 77030 USA

**Keywords:** Alternative splicing, IDX, H-ras, p19, p21, miRNAs, Costello syndrome, H-ras mutants

## Abstract

**Background:**

P19 H-Ras, a second product derived from the *H-Ras* gene by alternative splicing, induces a G1/S phase delay, thereby maintaining cells in a reversible quiescence state. When P21 H-Ras is mutated in tumour cells, the alternative protein P19 H-Ras is also mutated. The H-Ras mutation Q61L is frequently detected in different tumours, which acts as constitutive activator of *Ras* functions and is considered to be a strong activating mutant. Additionally, a rare congenital disorder named Costello Syndrome, is described as a *H-Ras* disorder in children, mainly due to mutation G12S in p19 and p21 H-Ras proteins, which is present in 90 % of the Costello Syndrome patients. Our aim is to better understand the role of p19 and p21 H-Ras proteins in the cancer and Costello Syndrome development, concerning the miRNAs expression.

**Methods:**

Total miRNAs expression regulated by H-Ras proteins were first analyzed in human miRNA microarrays assays. Previously selected miRNAs, were further analyzed in developed cell lines containing H-Ras protein mutants, that included the G12S Costello Syndrome mutant, with PCR Real-Time Taq Man miRNA Assays primers.

**Results:**

This study describes how p19 affects the RNA world and shows that: i) miR-342, miR-206, miR-330, miR-138 and miR-99b are upregulated by p19 but not by p19W164A mutant; ii) anti-miR-206 can restore the G2 phase in the presence of p19; iii) p19 and p21Q61L regulate their own alternative splicing; iv) miR-206 and miR-138 are differentially regulated by p19 and p21 H-Ras and v) P19G12S Costello mutants show a clear upregulation of miR-374, miR-126, miR-342, miR-330, miR-335 and let-7.

**Conclusions:**

These results allow us to conclude that the *H-Ras* G12S mutation plays an important role in miRNA expression and open up a new line of study to understand the consequences of this mutation on Costello syndrome. Furthermore, they suggest that oncogenes may have a sufficiently important impact on miRNA expression to promote the development of numerous cancers.

**Electronic supplementary material:**

The online version of this article (doi:10.1186/s12881-015-0184-z) contains supplementary material, which is available to authorized users.

## Background

*Ras*, an important family of proto-oncogenes in humans, consists of three members (*H-Ras*, *N-Ras* and *K-Ras*) located on human chromosomes 1, 11 and 12 respectively [1–3]. Indeed, *Ras* gene mutations have been implicated in up to 30 % of all tumours tested. These mutations are different and depend on the tissues involved but most commonly result in pancreatic (90 %) and colon and thyroid tumours (50 %), and lung and myeloid leukemias (30 %). A point mutation in *Ras* codons converts these genes into active oncogenes as a result of decreased GTPase activity, thereby restricting the easy interchange of GDP to GTP and resulting in a constitutive activation of the downstream pathways, or loss of GAP function. Ras mutated proteins are indirectly involved in metastasic phenotype development as they promote the acquisition of cumulative alterations in cellular pathways which result in cytoskeletal rearrangements, loss of cell adhesion (metalloprotease overexpression), tissue invasion, extravasation into lymphatic and blood vessels, and finally apoptosis evasion [4].

The *H-Ras* gene codifies for two different proteins, namely p19 H-Ras and p21 H-Ras, by alternative splicing [5–7]. The mRNA structures of these alternative sequences are identical in their first coding exons (called 1, 2, 3 and 4A) and have two non-codifying exons (0 and 4B located at the 5′-UTR and 3′-UTR regions, respectively) separated by intronic regions designated as A-E. The alternative exon, known as IDX (intron-D-exon, 82 nucleotides), is located between exons 3 and 4A. The pre-mRNA H-Ras is processed into two mRNAs, namely p21 mRNA, which excludes the IDX exon, and p19 mRNA, which includes it [5–7]. p19 mRNA therefore codes for a shorter protein than p21 mRNA. Furthermore, since IDX exon contains a premature stop codon, p19 does not contain the CAAX motif [6]. p19 H-Ras induces a G1/S phase delay, thereby maintaining cells in a reversible quiescence state [7]. p19 Binds to RACK1 and regulates telomerase activity upon interaction with p73α/β proteins as well as inducing hypophosphorylation of Akt and p70S6K and upregulating FOX1 [6, 7]. Although p19 overexpression does not induce apoptosis [7], Kim *et al.* have shown that it stimulates p73β-induced apoptosis when both proteins are simultaneously overexpressed [8]. RNAi of p19 increases cell growth, thereby having an opposite effect to the delay in the G1/S phase described above [7]. Other authors showed that p19 represses proliferation on non-small cell lung cancer through interaction with neuron-specific enolase (NSE) [9].

P19 was first described from in T24/EJ bladder carcinoma cell line that contains, in addition to G12V mutation, a small number of other nucleotide changes including an adenine (A) to guanine (G) mutation at position 2714. This latter region was showed to regulate the alternative splicing of H-Ras into two proteins, p21 and p19. The 2719 mutation decresed the p19 expression, and this mutation promotes 10-fold increase of p21 H-Ras levels and a corresponding increase of the transforming efficiency of structurally activated alleles [5, 10].

The H-Ras mutation Q61L is frequently detected in different tumour cell lines, where it acts as a constitutive activator of the Ras-signalling pathway and is considered to be a strong activating mutant by decreasing GTPAse activity and increasing GDP/GTP exchange [1, 11, 12]. G12S, another important *H-Ras* mutation is, present in more than 90 % of patients with Costello Syndrome (CS), a rare congenital disorder caused by germ-line activation of *H-Ras* oncogenes that affects both p19 and p21 H-Ras [13]. CS is characterized by severe failure-to-thrive, cardiac abnormalities, including tachyarrhythmia and hypertrophic cardiomyopathy, a predisposition to papillomata and malignant tumours, and neurologic abnormalities, including nystagmus, hypotonia developmental delay, and mental retardation [14–18].

To better understand the role of p19 and p21 H-Ras proteins in the development of cancer, we transfected HeLa cells with p19 and p21 mutant sequences, which were reported in the literature to be commonly detected in tumour cell lines and in CS (G12S). We also evaluated the expression of selected miRNAs [19] involved in some aspects of metastasis and others related with aggressive small cell lung cancer.

## Methods

### Cell lines, cell transfection and antibodies

HeLa cells were cultured and transiently transfected as described elsewhere [6, 7]. Knock-out murine embryonic fibroblasts (MEFs) H-Ras^−/−^ (KO) and double knock-out MEFs H-Ras^−/−^ plus N-Ras^−/−^ (DKO) were obtained from Dr. E. Santos’s laboratory [20]. KO and DKO cell lines, stably expressing pEGFP (negative control), pEGFP-p19 and pEGFP-21, were obtained by transfecting the fibroblasts with the specific vector and selecting with geneticin.

### Plasmids

pEGFP-p19 and -p21, pRK5-p19 and pRK5-p19W164A have been described previously [6, 7], therefore the other pRK5 plasmids were obtained in a similar manner [6, 7]. Other point mutations of p19 H-Ras and p21 H-Ras were performed by polymerase chain reaction site-directed mutagenesis using the QuickChange® Site-Directed mutagenesis kit from Stratagene.

### Isolation of small RNAs

miRNAs were extracted using the miRVANA™ miRNA isolation kit from Ambion Inc. (Austin, Tx). Isolation was performed as described by the manufacturer’s protocol.

### miRNA microarrays

miRNA microarrays were performed as described previously [21].

### miRNA Reverse Transcriptase (RT) reaction

cDNA was reverse-transcribed from enriched miRNA samples (miRVANA kit) using specific miRNA primers from the Taq Man MicroRNA Assay and reagents from the Taq Man MicroRNA RT kit (AB Applied Bio systems) according to the manufacturer’s instructions. Briefly, 1.33 μL of each resulting cDNA was amplified by PCR using Taq Man MicroRNA Assay primers with the Taq Man Universal Non amperase PCR Master Mix (in a total volume of 20 μL) and analyzed with a 7500 ABI PRISM Sequence Detector System according to the manufacturer’s instructions. miRNA expression was calculated from the relevant signals by normalization with respect to the signal for U18 (for HeLa cells) and U6 for MEFs. Stem-loop quantitative RT-PCR for mature miRNAs was performed as described previously using an Applied Biosystems ABI 7500 Real Time PCR system. All RT-PCR reactions were run in triplicate and gene expression, relative to U18, calculated using the 2-ΔΔCt method [22].

### Real time TAQMAN RT-PCR assays of mRNAs

Total RNA was extracted from 10 × 10^5^ HeLa cells using TRIZOL reagent (Life Technologies, Inc.), as described previously. cDNA was reverse-transcribed from total RNA samples using SuperScriptIII® from Invitrogen. The resulting cDNA was amplified by PCR using Taq Man Assay primers with the Taq Man Universal Non-amperase PCR Master Mix and analyzed with a 7500 ABI PRISM Sequence Detector System according to the manufacturer’s instructions. mRNA expression was calculated from the relevant signals by normalization with respect to the signal for glyceraldehyde-3-phosphate dehydrogenase (GAPDH) mRNA expression. The assay numbers for exons E3-IDX p19 H-Ras (Hs00978053_g1), E4A-E4B H-Ras total (Hs00978051_g1) and E3-E4A p21 H-Ras (Hs00610483_m1) and for GAPDH (HS99999905_m1 housekeeping) were supplied by Applied Biosystems Gene Expression Assays (Applied Biosystems). Assays were run with Taqman Universal.

### Western blot analysis, cell-proliferation assay and determination of cell-cycle phase percentages

These assays were performed as described previously [6, 7].

### Ethics approval

No ethics approval was required for aspects of this study.

## Results

### P19 overexpression regulates specific miRNA expression

**Fig. 1:**
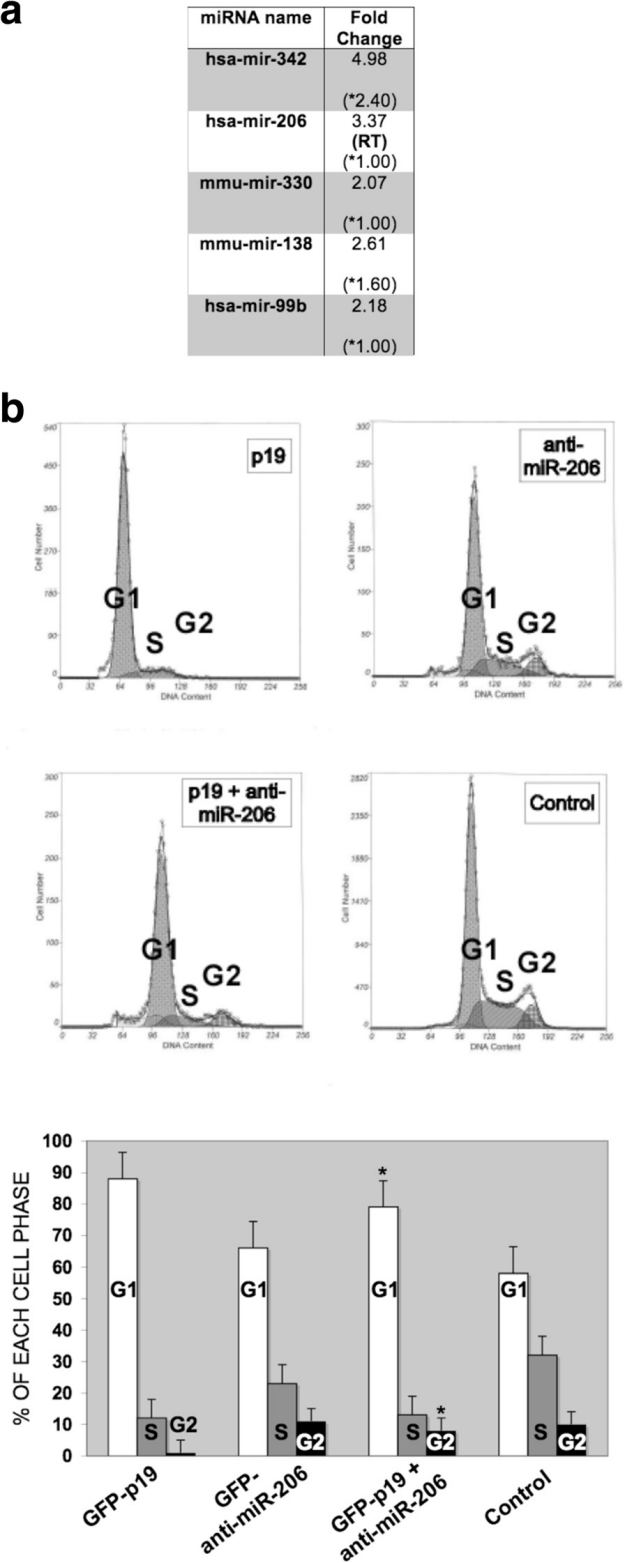
Anti-miR-206 partially antagonizes the effect of p19 on G1/S and G2 phases. **a** Fold change is the value used to measure overexpression of pRK5-p19 as compared first to pRK5 empty vector and then to overexpression of pRK5-p19mut for miRNA microarrays. (*) indicates the value obtained with pRK5-p19mut. These miRNAs varies upon overexpression of 19 and not by p19mut overexpression. Additional gene information is available in Additional file 1 (*P* < 0.01). RT: miR-206 activation was confirmed by Taqman RT-PCR with a specific Taqman assay for mature miR-206 (Applied Biosystems). **b** HeLa cells were first transfected with the pSuper-GFP vector containing the sequence of anti-miR-206 and incubated for 2 days under cell-culture conditions. Cells were then re-transfected with GFP-p19 vector and analyzed by FACS (fluorescent-activated cell sorting) the following day. A representative flow cytometry histogram with GFP-p19, pSuper-GFP anti-miR-206, GFP-p19 + pSuper-GFP anti-miR-206 or GFP only (negative control) is shown in the upper portion, whereas G1, S, and G2 percentages are given below (*P < 0.001 as compared G1 and G2 to GFP-19 versus GFP-p19 + anti-miR-206). HeLa cells line were used in all the experiments stated above

We have shown previously that cell growth and metastatic genes are regulated by p19 [7]. In this latter work we compared p19 expression with the specific p19mut expression (mutation not seen in p21), where p19mut was a p19 mutant (W164A) that prevented p19-RACK1 and p19-p73α binding [7]. These previous analysis are further complemented here by checking whether p19 can regulate other RNA genes by studying miRNA expression in cells overexpressing p19 or p19mut. First, we incubated miRNA micro-arrays with RNAs from cells overexpressing p19 wild-type protein *versus* RNAs from cells overexpressing empty vector. A second incubation was performed with RNAs from cells overexpressing p19mut in a similar way. Afterwards, both results were mathematically compared, and miRNAs that are upregulated by p19 overexpression but not by 19mut overexpression were selected (see Fig. 1a). Candidate miRNAs were found to be miR-342, miR-206, miR-330, miR-138, and mirR-99b (Fig. 1a and Additional file 1), which vary with p19 but not with the specific p19mut overexpression. We consider that the values obtained with p19mut overexpression could be considered the miRNAs basal levels respect p19 wild-type protein (see asterisks values in Fig. 1a and Additional file 1). The most relevant observation of this assay is that the expression of very few miRNAs varies upon overexpression of p19 and not by p19mut overexpression and so, these miRNAs might be also related to the pathway process activated by RACK-p19 and p73α-p19 interactions. miRNA upregulation by p19 H-Ras was re-validated by RT-PCR with a specific Taqman assay for mature miR-206, miR-342, miR-138 and miR-330 and was found to increase 2-, 1.6-, 16- and 2.5-fold, respectively, upon overexpression of p19 in three independent experiments and quadruplicate analysis. miR-206 has been reported to be the miRNA whose expression is most downregulated in metastatic cells and has also been shown to regulate cell migration and morphology [23]. Our previous work showed that p19 H-Ras induces a G1/S phase delay, thereby maintaining cells in a reversible quiescence state [7]. Taken together, these findings prompted us to study the contribution of miR-206 upregulation to the G1/S delay, both of which are induced by p19. Figure 1b shows that anti-miR-206 partially antagonizes the effect of p19 on G1/S phases and restores the G2 phase, thus indicating that miR-206 upregulation partially contributes to the G1/S delay observed upon p19 overexpression.

### H-Ras mutants alter the H-Ras splicing rate

**Fig. 2:**
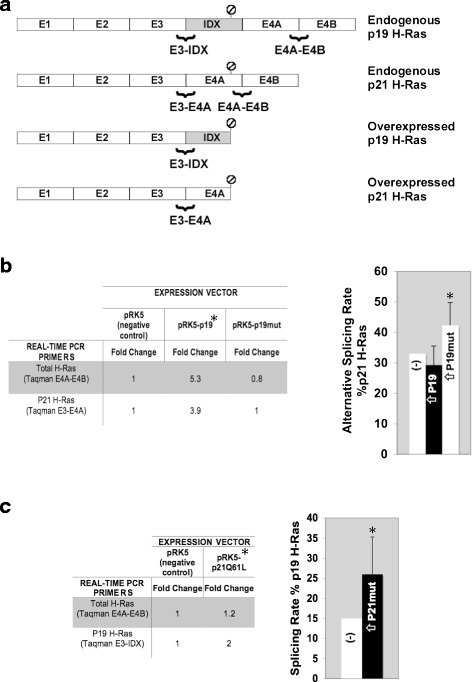
Gene expression and alternative splicing regulation by H-Ras proteins. **a** Scheme of the Taqman assays used to determine endogenous p19, p21, and endogenous total H-Ras mRNA expression. E3-IDX, E3-E4A, and E4A-E4B recognize endogenous p19 H-Ras, p21 H-Ras, and both p19 and p21 H-Ras, respectively. Taqman assays were performed with E3-E4A and E4A-E4B for overexpressed p19 and with E3-IDX and E4A-E4B for overexpressed p21mut. **b** Endogenous total H-Ras (Taqman E4A-E4B) and p21 (Taqman E3-E4A) mRNA levels change upon overexpression of p19 and p19mut. The results are presented as fold changes with respect to basal levels obtained for empty vector transfections, which were set to 1 (no change). Graphic: the percentage of alternative splicing p21/endogenous total H-Ras (Taqman E3-E4A/Taqman E4A-E4B) was obtained for each individual experiment and standard deviations of three separate experiments calculated. (−) is the % p21 in transfections with empty vector set to 33 %; (⇧) p19 and (⇧) p19mut, % is the p21 in HeLa cells overexpressing p19 and p19mut, respectively. (⇧) p19, *P* = 0.07; (⇧) p19mut, *P* = 0.08. **c** Endogenous total H-Ras (Taqman E4A-E4B) and p19 (Taqman E3-IDX) mRNA level changes upon overexpression of p21Q61L. The results are presented as fold changes with respect to the basal levels obtained for empty vector transfections, which were set to 1 (no change). Graphic: the percentage of alternative splicing p19/total H-Ras (Taqman E3-IDX/Taqman E4A-E4B) was obtained for each individual experiment and standard deviations of three separate experiments calculated. (−) is the % of p19 in transfections with empty vector was set to 15 %; (⇧)p21mut is the % of p19 after expression of the p21mut (p21Q61L). *P* = 0.09. Hela cells line were used in all the experiments stated above

We have previously demonstrated that SR proteins (SFRs) activate inclusion of the alternative exon IDX and subsequently p19 expression [6, 24]. p68 RNA helicase (DDX5) was also shown to inhibit p19 expression and reduce IDX inclusion, whereas SC-35 (SFRS2) and SRp40 (SFRS5) strongly increase IDX inclusion *in vivo* [6, 24]. We analyzed the obtained miRNAs by the TARGETSCAN tool and observed that miR-330 may target SC-35 and many other SR proteins as well as an UsnRNP core protein, mir-342 may target SF4, and miR-206 may target p68 RNA helicase and many other SR proteins. As splicing factors are also targets of the miRNAs upregulated by p19, therefore we decided to study whether the overexpression of p19 can also affect its own alternative splicing. Thus, two Taqman RT-PCR assays were designed to map total/endogenous H-Ras mRNA (directed to E3-E4A exons) and the endogenous p19 level and the endogenous/total p21 H-Ras ratio determined in HeLa cells transiently overexpressing p19 (see Fig. 2a). Figure 2b shows that overexpression of p19 increases total endogenous H-Ras and endogenous p21 expression by 5.3- and 3.9-fold, respectively, and that this activation is reverted to endogenous basal levels by p19mut. The splicing rate for endogenous p21 formation was slightly reduced in cells overexpressing p19 (Fig. 2b) but increased in the presence of p19mut (10 % higher than the negative control), thus indicating that p19mut can alter the p19:p21 ratio by acting on the H-Ras alternative splicing processes. In contrast, p19 was observed to directly regulate total mRNA H-Ras expression but not to significantly alter the splicing rate of H-Ras pre-mRNA, thus indicating that the p19:p21 ratio is maintained. Furthermore, we showed that the overexpression of the strong p21 mutant (p21Q61L) also regulated this alternative splicing, increasing the amount of p19 mRNA (Fig. 2c).

### P19 alters miRNAs expression but not the cell growth in H-Ras^(−/−)^ KO cell lines

**Fig. 3:**
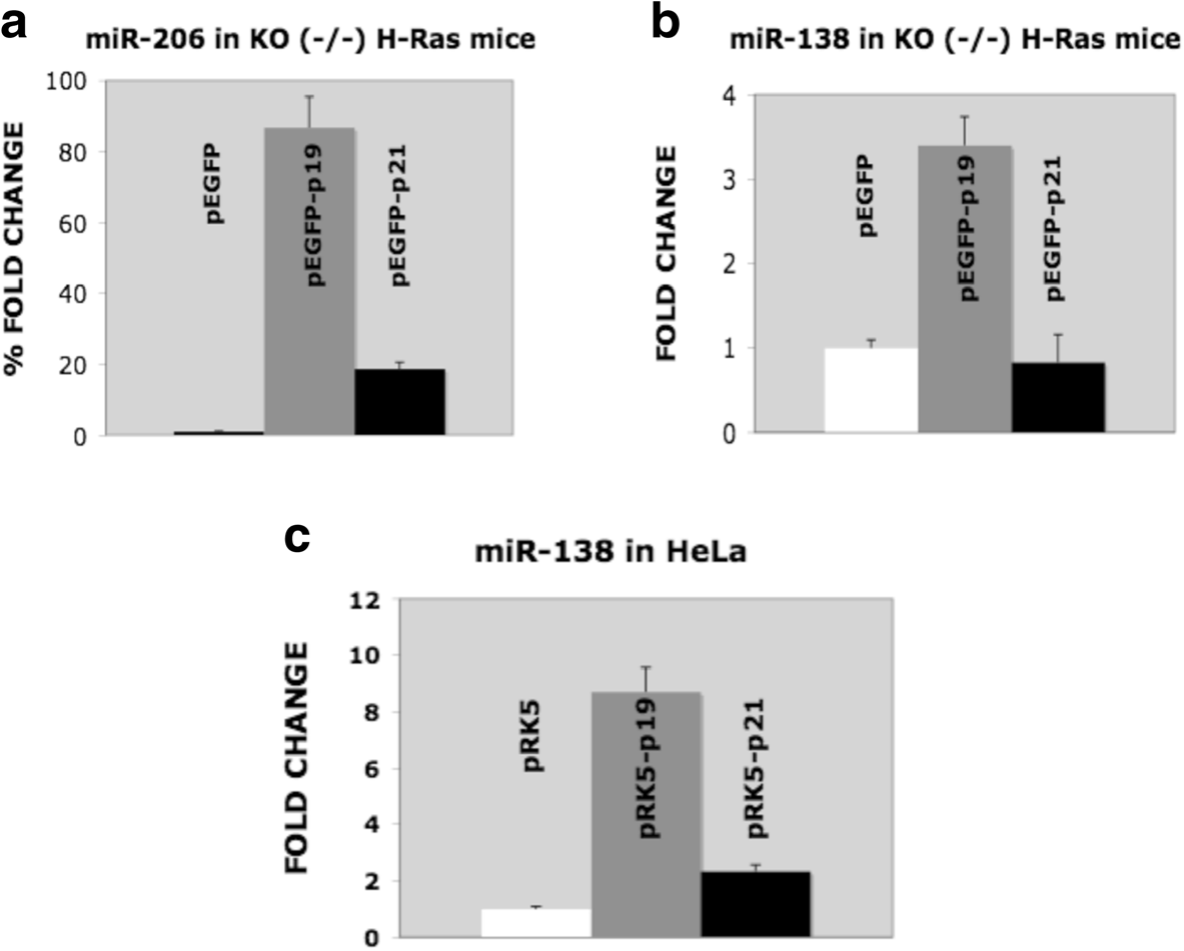
P19 and p21 H-Ras differentially regulate miR-206 and miR-138. **a** and **b** show KO H-Ras^(−/−)^ fibroblasts stably expressing pEGFP (negative control), pEGFP-p19 or pEGFP-p21. The regulation of miR-206 (**a**) and miR-138 (**b**) was analyzed in these cells with specific miRNA Taqman assays. **c** Regulation of miR-138 in transient transfections in HeLa cells

**Fig. 4:**
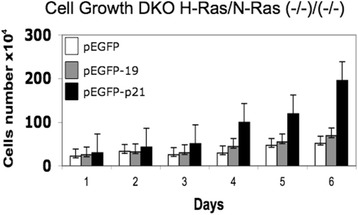
P19 H-Ras does not activate cell growth. DKO H-Ras^(−/−)^/N-Ras^(−/−)^ fibroblasts that stably express pEGFP (negative control), pEGFP-p19 and pEGFP-p21 were studied by direct cell-proliferation assay. Cells were harvested and plated in 96 wells (10,000 cells/well) on six microplates in sextuplicate and incubated at 37 °C, 5 % CO_2_ with DMEM/10 % FCS. After the desired time, the microplates were washed and frozen. Cells were quantified with the green fluorescent dye CyQuant (Invitrogen) according to the manufacturer’s instructions. Fluorescence measurements were performed using a microplate reader with excitation at 485 nm and detection at 530 nnm

In order to further understand how the alternative splicing of H-Ras alters miRNAs, we differentially expressed pEGFP-p19 or pEGFP-p21 in H-Ras^(−/−)^ KO MEFs and analyzed their effect on miR-206 and miR-138. Figure 3a shows that p19 has a large effect on miR-206 expression whereas pGFP-p21 increases it only slightly. pEGFP-p19 also has a large effect on miR-138 in both KO MEFs and HeLa cells (Fig. 3b, c, respectively), thus indicating that the alternative splicing of H-Ras towards p19 or p21 affects the expression of several miRNAs. Figure 4 shows that pEGFP-p21 increases cell growth in H-Ras^(−/−)^/N-Ras^(−/−)^ DKO MEFs whereas pEGFP-p19 does not. This finding is in accordance with our previous results, where we showed that p19 causes a G1/S delay in HeLa cells.

### The p19 CS mutant G12S alters miRNA expression

**Fig. 5:**
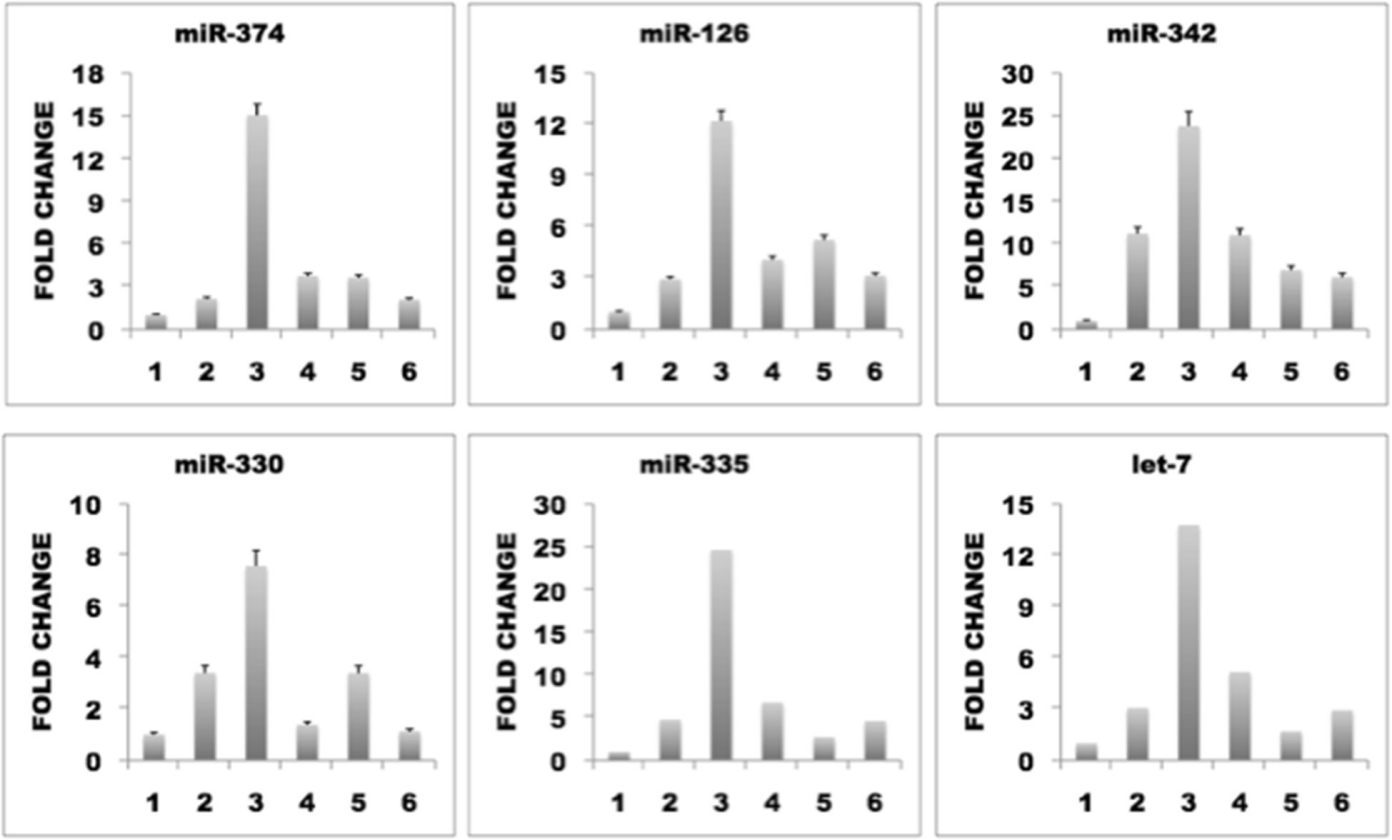
P19 H-RasG12S CS mutant clearly upregulates several miRNAs. HeLa cells transiently expressing: 1) pRK5 (negative control); 2) pRK5-p19; 3) pRK5-p19G12S; 4) pRK5-p21; 5) pRK5-p21Q61L; and 6) pRK5-p21G12S. MiRNAs levels were analyzed with specific miRNAs Taqman assays. Three independent experiments were done, each of them per quadruplicate (*n* = 12, and statistic significative difference calculated by Anova, one way analysis of variance, *p* <0.05, *p* value = 0.036), GraphPad Prism version 4.0

Aoki *et al*. [25] reported that CS is a result of mutations in the *H-Ras* gene. The most common mutation is found on codon 12 with the amino acid change G12S [13]. All human cells contain both p19 and p21 H-Ras proteins at different levels as a consequence of the alternative splicing, therefore when *H-Ras* gene is mutated in CS patients, both these proteins p19 and p21, are present in their mutated forms. We obtained pRK5 plasmids containing p19(G12S) and determined how this mutant alters a selected group of miRNAs in HeLa cells. These miRNAs included miR-342 and miR-330, which are already known to be upregulated by p19 overexpression (Fig. 1), miR-126 and miR-335, which significantly reduce the ability of CN34-LM1 and CN34-BoM1 cells to metastasize to the lung [23], miR-374, which is known to be associated with aggressive small cell lung cancer [26], and let-7, which targets *Ras* genes [27]. Figure 5 shows that p19(G12S) clearly upregulates all these miRNAs (column 3), thus indicating that the p19 CS mutant contributes significantly to the syndrome by altering specific miRNA levels. We also analyzed how p21 mutants affect the same set of miRNAs by overexpressing PRK5-p21, pRK5-p21Q61L and pRK5-p21G12S in HeLa cells. Figure 5 shows that p21 and p21 mutants also alter expression of these miRNAs, although to a lesser extent than p19G12S. Thus, p21 was found to upregulate miR-374, miR-126, miR-342, miR-335 and let-7 but not miR-330, and p21G12S was found to have the same effect as p21 on miR-374, miR-342 and miR-126. In contrast, p21G12S (column 5) was found to have no effect on miR-335 and let-7 but upregulates miR-330, whereas p21 does not. This indicates that p21 and p21 mutants do not have such a linear effect as p19 and p19G12S but that both p19 and p21 CS mutants alter the expression of several miRNAs, which may contribute to the development of CS.

## Discussion

### Homeostatic regulation of H-Ras

Although p21 H-Ras mutants have been extensively studied, this is not the case for specific p19 H-Ras mutants. However, it is known that a mutation in the 5′ splice-site of the IDX exon of *H-Ras* (position 2719, A to G change) induces a 10-fold increase in p21 H-Ras level and a corresponding increase in the transforming activity in a bladder carcinoma cell line [10]. In addition, we have previously reported that the mouse NIH 3 T3 cell contains a mutation at position 165 which causes a D to G change [6]. p19 overexpression activates full-length total H-Ras and p21, although the endogenous p19:p21 ratio due to alternative splicing remains relatively unchanged (Fig. 2). This H-Ras and p21 activation is dependent on the W164A amino acid (Fig. 2). Thus, whereas the specific p19mut expression reverts endogenous total H-Ras to basal levels, p19mut alters alternative splicing in favor of p21. In this study, we also found that although the Q61L mutation in p21 does not affect endogenous total H-Ras expression, the Q61L mutation on p21 produces a clear change in the p19:p21 ratio by doubling the amount of p19 mRNA compared to wild-type. We can therefore conclude that the observed altered alternative splicing may act as a homeostatic response mechanism. We have also found that few proteins or miRNA genes are up- or downregulated when p19 is overexpressed (but not with the specific p19mut overexpression), showed in this work and [7]. Indeed, the response mechanisms described in this work when p19, p19mut or the p21 mutant are overexpressed correlate well with observations in *H-Ras* knockout (KO) mice. These KO mice were found to be viable [28] and fibroblasts obtained from them had few genes that modified their expression [20]. These findings indicate that a cellular response mechanism is in place that overcomes the lack of *H-Ras*, thereby allowing the mice to survive, and that a strong homeostatic mechanism plays a role in controlling p21 and p19 levels, as p21 drives cell proliferation whereas p19 maintains a reversible cellular quiescence state [7].

### The role of p19 in cancer and metastasis processes

It may be significant that H-Ras is regulated by miRNAs, such as the let-7 miRNA family [27, 29], and that the alternative splicing of H-Ras, which favors p19 over p21, has consequences as regards the levels of certain miRNAs (Fig. 1a and Additional file 1). Two of the miRNAs upregulated by p19 (Additional file 1) have previously been reported to be of significant interest in cancer studies: miR-342 is one of the miRNA markers for acute promyelocytic leukemia [30, 31] and miR-206 suppresses ERα in breast cancer cell lines and also plays a role in muscular dystrophy [32–34]. miR-335, miR-206 and miR-126 have been shown to significantly reduce the ability of certain cells to metastasize to the lung [23, 35]. These latter results have driven us to further study the regulation of miR-206 by H-Ras proteins as overexpression of p19 causes G1/S delay [7] and upregulates miR-206 (Fig. 1a). We are aware of the limitations of those studies performed with overexpression of a CS mutant. Having this point in mind, we presented here experiments with KO H-Ras^(−/−)^ DKO H-Ras^(−/−)^/N-Ras^(−/−)^ that stably express p21 or p19. Additionally, we obtained some preliminary results of miRNAs Taqman RT-PCR expression profile of G12S or G12A endogenous p19 and p21 proteins in fibroblasts cell lines established from CS tumour patients. Those results showed that miR-330, miR-335 and miR-374 are statiscaly and significally overexpressed in those CS patients cells, and thus they are putative cadidates to be miRNAs overexpressed in CS patients (R. García-Cruz and K. Sol-Church, personnel communication). Herein we have shown that combining p19 overexpression with a miR-206 inhibitor results in a partial decrease of the G1 phase with a clear recovery of the G2 phase, thus indicating that miR-206 is one of the factors contributing to the delay of the G1/S phase. Additionally, we have shown that miR-206 is regulated by the alternative splicing of H-Ras (Fig. 3a) as the ovexpression of p19 upregulates miR-206 more effectively than p21 H-Ras when pEGPP-19 and pEGPP-21 are stably expressed in KO H-Ras^(−/−)^. miR-138, a miRNA that suppresses invasion and promotes apoptosis in some carcinoma cells [36], was also studied. Figure 3b, c show that miR-138 is clearly upregulated in KO H-Ras^(−/−)^ mice that stably express pEGPP-19, whereas it is unaffected in KO H-Ras^(−/−)^ mice that stably express pEGPP-21 (Fig. 3b). This observation was corroborated by the transient expression of both proteins (Fig. 3c). Finally, we also corroborated our previous studies in which we demonstrated that p19 H-Ras does not induce growth using DKO H-Ras/N-Ras^(−/−)/(−/−)^ mice that stably express pEGPP-19 (see Fig. 4). As we showed here that miR-206 regulates cell growth (being these observations in agreement with previous published results, see below) we discuss here our a putative protein 3′-UTR target that could be having a role on these cell growth regulation. Adams *et al.* have indentified ERα as a direct miR-206 target, and further demonstrated that miR-206 inhibited the mRNA and protein expression of ERα in human ovarian cells [32]. Additionally, expression of miR-206 has been showed to inhibit cellular proliferation and to disturb invasion in ERα–positive endometrial carcinoma cells [37].

## Conclusions

H-Ras mutants have been described as a potential marker for CS [15, 38, 39], although the mutations in IDX sequences and the rasISS1 splicing silencer described in [24] have not been observed in several patients with this CS (K. Sol-Church, personnel communication). This finding strongly suggests that H-Ras-related mutations in CS are likely to be found in the common amino acid sequences and thereby affect the complementary functions of p21 and p19. We can therefore conclude that both, p21 and p19, must malfunction in order for a subject to develop CS. Around 90 % of CS patients have the G to A mutation that results in the G12S amino acid change. We therefore tested how this mutation affects a selected group of cancer-related miRNAs and found a significant upregulation by p19 H-Ras G12S in all cases. This allowed us to conclude that the H-Ras G12S mutation plays an important role in miRNA expression and therefore opens up a new line of study to understand the consequences of this mutation on CS. Furthermore, this finding has further consequences for many cancers as our results indicate that oncogenes may have a sufficiently important impact on miRNA expression to promote their development.

### Availability of supporting data

Additional file 1 include an additional table. Microarray data accession number: ETABM-494.

## Additional file

Additional file 1:
**miRNAs upregulated by p19-overexpression but downregulated by p19mut-overexpression.**
