# Supplementary material for: The role of p19 and p21 H-Ras proteins and mutants in miRNA expression in cancer and a Costello syndrome cell model
Source: BMC Med Genet. 2015 Jul 3;16:46. doi: 10.1186/s12881-015-0184-z (PMC4631104; doi:10.1186/s12881-015-0184-z)
Supplement: Additional file 1: — miRNAs upregulated by p19-overexpression but downregulated by p19mut-overexpression. [file 12881_2015_184_MOESM1_ESM.pdf]

Additional File 1

miRNAs upregulated by p19-overexpression but downregulated by p19mut-overexpression

miRNA name	Fold Change	Selected Target Genes
**hsa-mir-342**	4.98 (*2.40)	***BCL2L1, BCL2L2, CSK, FOSB, IBRDC2, MAP3K7IP2, RAP2B, RASA1, RASSF1, SF4, SLA***
**hsa-mir-206**	3.37 **(RT)** (*1.00)	***ARHGAP21,CDK6, CDK9, DDX5, FOSB, JUND, MAP3K3, MET, MTSS1, RASA1, SFRS1, SFRS10, SFRS3, SFRS3. SFRS7, SFRS9,SFS7***
**mmu-mir-330**	2.07 (*1.00)	***CDC42, CDK11, DICER1, E2F1, GAS7, MAP2K1, PIK3R1, PRKCA/PKCα, PRKCB1/PKCβ1, PRKCE/PKCε, SFRS1, SFRS10, SFRS2, SFRS7, SFRS8, SFRS9, SLITRK4, SNRPD3***
**mmu-mir-138**	2.61 (*1.60)	***AIFM2, CCND3, CCNE1, DDX3X, MAP2K7,TCTP, MAPK4, MYBBP1A, TP53BP2, TP53INP1, TP53INP2, TP73L/p63***
**hsa-mir-99b**	2.18 (*1.00)	***MYCBP2, RalGPS1A***

Fold change is the value measuring overexpression of pRK5-p19 as compared first to pRK5 empty vector and then compared to overexpression of pRK5-p19mut for miRNAs microarrays. (*) indicates the value obtained with pRK5-p19mut. Then, data show miRNAs that are upregulated by p19-overexpression and, additionally, downregulated by p19mut-overexpression. Gene mRNA targets are described in http://microrna.sanger.ac.uk/sequences/ by using MIRANDA; TARGETSCAN, and PICTAR-VERT. (P<0.01). RT: mir-206 activation was confirmed by Taqman Real-PCR with an specific Taqman assay for mature miR-206 (Applied Byosystems).

Functions of the Selected Target Genes:

hsa-miR-342

BCL2L1	ENSG00000171552	apoptosis regulator Bcl-X (Bcl-2-like 1 protein); apoptosis inhibitor.
BCL2L2	ENSG00000129473	apoptosis regulator Bcl-W (Bcl-2-like 2 protein); apoptosis inhibitor.
CSK	ENSG00000103653	c-src tyrosine kinase
FOSB	ENSG00000125740	Protein fosB,G0/G1 switch regulatory protein 3
IBRDC2	ENSG00000137393	p53-inducible RING finger protein.
MAP3K7IP2	ENSG00000055208	mitogen-activated protein kinase kinase kinase 7 interacting protein 2
RAP2B	ENSG00000181467	Ras-related protein Rap-2b
RASA1	ENSG00000145715	Ras GTPase-activating protein 1,Ras p21 protein activator.
RASSF1	ENSG00000068028	Ras association (RalGDS/AF-6) domain family 1. RASSF1A functions as a negative regulator of cell proliferation through inhibition of G1/S-phase progression.
SF4	ENSG00000105705	splicing factor 4
SLA	ENSG00000155926	Src-like-adapter protein 1

hsa-miR-206

ARHGAP21	ENSG00000107863	Rho GTAase activating protein p21;
CDK6	ENSG00000105810	cyclin depenent kinase 6
CDK9	ENSG00000136807	cyclin-dependent kinase 9
DDX5	ENSG00000108654	p68 RNA helicase
FOSB	ENSG00000125740	Protein fosB (G0/G1 switch regulatory protein 3)
JUND	ENSG00000130522	Jun D proto-oncogene,Transcription factor jun-D.
MAP3K3	ENSG00000198909	mitogen activated kinase 3
MET	ENSG00000105976	met proto-oncogene (hepatocyte growth factor receptor)
MTSS1	ENSG00000170873	metastasis suppressor 1.
RASA1	ENSG00000145715	Ras GTPase-activating protein

RASA1	ENSG00000145715	Ras GTPase-activating protein 1,Ras p21 protein activator.
SFRS1	ENSG00000136450	Splicing factor, arginine/serine-rich 1, SR protein, (pre-mRNA splicing factor SF2) (Alternative splicing factor ASF-1)
SFRS10	ENSG00000136527	Arginine/serine-rich splicing factor 10, SR protein HTRA2-beta
SFRS3	ENSG00000112081	Splicing factor, arginine/serine-rich 3, SR protein SRP20
SFRS7	ENSG00000115875	splicing factor, arginine/serine-rich 7, SR protein 9G8
SFRS9	ENSG00000111786	splicing factor, arginine/serine-

hsa-miR-330

GENE TARGETS	ENSEMBL NUMBER	FUNCTION
CDC42	ENSG00000070831	Cell division control protein 42 homolog
CDK11	ENSG00000155111	Cyclin-dependent kinase (CDC2-like) 11
DICER1	ENSG00000100697	Endoribonuclease Dicer Helicase with RNase motif.
E2F1	ENSG00000101412	Transcription factor E2F1
GAS7	ENSG00000007237	Growth-arrest-specific protein 7
MAP2K1	ENSG00000169032	MAPK/ERK kinase 1
PIK3R1	ENSG00000145675	PI3-kinase p85-alpha subunit.
PRKCA/PKCa	ENSG00000154229	Protein kinase C, alpha type
PRKCB1/PKCb1	ENSG00000166501	Protein kinase C, beta type
PRKCE/PKCe	ENSG00000171132	Protein kinase C, epsilon type
SFRS1	ENSG00000136450	Splicing factor, arginine/serine-rich 1, SR protein, (pre-mRNA splicing factor SF2) (Alternative splicing factor ASF-1)
SFRS10	ENSG00000136527	splicing factor, arginine/serine-rich 10, SR protein, TRA2
SFRS2	ENSG00000161547	Splicing factor, arginine/serine-rich 2, SR protein SC35
SFRS7	ENSG00000115875	splicing factor, arginine/serine-rich 7, SR protein 9G8
SFRS8	ENSG00000061936	splicing factor, arginine/serine-rich 8, SR protein (suppressor-of-white apricot homolog, Drosophila, SWAP)
SFRS9	ENSG00000111786	Splicing factor, arginine/serine-rich 9, SR protein SRp30, (Pre-mRNA splicing factor SRp30C

SLITRK4	ENSG00000179542	slit and trk like 4 protein; slit and trk like gene 4
SNRPD3	ENSG00000100028	Small nuclear ribonucleoprotein Sm D3 (UsnRNP core protein D3) Splicing factor

miR-138

AIFM2	ENSG00000042286	apoptosis-inducing p53-responsive gene
CCND3	ENSG00000112576	G1/S-specific cyclin D3.
CCNE1	ENSG00000105173	G1/S-specific cyclin E1.
DDX3X	ENSG00000124487	DEAD-box protein 3 (Helicase-like protein 2)
MAP2K7	ENSG00000076984	mitogen-activated protein kinase kinase 7,JNK kinase 2, c-Jun kinase 2) (JNK kinase 2)
MAPK4	ENSG00000141639	Mitogen-activated protein kinase 4, ERK-4, MAP kinase isoform p63
MYBBP1A	ENSG00000132382	MYB binding protein 1a; p53-activated protein-2
TP53BP2	ENSG00000143514	Apoptosis stimulating of p53 protein, p53-binding protein 2).
TP53INP1	ENSG00000164938	tumor protein p53 inducible nuclear protein 1
TP53INP2	ENSG00000078804	protein p53 inducible nuclear protein 2
TP73L/p63	ENSG00000073282	tumor protein p73-like; tumor protein63 kDa with strong homology to p53.

miR-99b

MYCBP2	ENSF00000004253	MYC binding protein 2
RalGPS1A	ENSG00000136828	Similar to Ral guanine nucleotide exchange factor
